# Deep Learning-Based Artistic Inheritance and Cultural Emotion Color Dissemination of Qin Opera

**DOI:** 10.3389/fpsyg.2022.872433

**Published:** 2022-04-21

**Authors:** Han Yu

**Affiliations:** ^1^School of Journalism and Communication, Northwest University, Xi’an, China; ^2^Apparel and Art Design College, Xi’an Polytechnic University, Xi’an, China

**Keywords:** Qin opera, deep learning, emotion, attention model, residual network

## Abstract

How to enable the computer to accurately analyze the emotional information and story background of characters in Qin opera is a problem that needs to be studied. To promote the artistic inheritance and cultural emotion color dissemination of Qin opera, an emotion analysis model of Qin opera based on attention residual network (ResNet) is presented. The neural network is improved and optimized from the perspective of the model, learning rate, network layers, and the network itself, and then multi-head attention is added to the ResNet to increase the recognition ability of the model. The convolutional neural network (CNN) is optimized from the internal depth, and the fitting ability and stability of the model are enhanced through the ResNet model. Combined with the attention mechanism, the expression of each weight information is strengthened. The multi-head attention mechanism is introduced in the model and a multi-head attention ResNet, namely, MHAtt_ResNet, is proposed. The network structure can effectively identify the features of the spectrogram, improve the weight information of spectrogram features, and deepen the relationship between distant information in long-time series. Through experiments, the proposed model has high emotional classification accuracy for Qin opera, and with the increase of the number of data sets, the model will train a better classification effect.

## Introduction

There are various forms and varieties of Chinese traditional operas, among which Qin opera has the longest history. It was formed in the Western Zhou dynasty and gradually developed and matured after the Qin Dynasty (Zhen, 2006; Li and Wang, 2016; Li, 2018; Zhang, 2018). At present, it has become one of the important traditional operas in China. Qin opera is based on the folk songs and dances of the people in Shaanxi and Gansu. It has strong local characteristics and local elements. The local people perform casually with a high voice in their daily life and work and express their true emotional state through singing.

At present, the expression form of folk art is too single and lacks innovation. The young generation pays less and less attention to opera, and the traditional folk skills have faded out of people’s lives, which will be a severe challenge to the inheritance of Qin opera. In order to attract more young people’s attention, we can consider integrating more modern elements into the performance forms of Qin opera. The new performance forms will give Qin opera new vitality and life so that Qin opera can be better carried forward and inherited. For example, integrating virtual reality technologies, such as Virtual Reality (VR) display or immersive experience into the performance of traditional Qin opera, displaying Qin opera in multiple ways, and realizing the integration of traditional opera and modern elements will bring completely different feel to the audience. In the display, the transformation of each scene and the expression of drama emotion need to be recognized by the computer (She, 2006; Liu and Wang, 2008; Wang, 2010; Liu, 2014; Zhang J. et al., 2018). How to enable the computer to accurately analyze the emotional information and story background of characters in Qin opera is a problem that needs to be studied. Therefore, a specific study on the automatic emotion recognition of Qin opera will be carried out, so as to promote the artistic inheritance and cultural emotion color dissemination of Qin opera.

The emotion of Qin opera is complex and diverse, and it is difficult for computers to understand it automatically. Therefore, how to make the computer automatically recognize the emotion expressed in Qin opera is an urgent problem to be solved. At present, deep learning technology is widely used in audio processing-related fields. However, the existing models are not accurate enough to classify long-time series data. Therefore, how to improve the accuracy of the model in Qin opera emotion recognition is the main research content of this paper.

## Related Works

At present, with the development of artificial intelligence, deep learning technology is widely used in audio processing-related fields. Song et al. (2014) proposed a feature extraction generation model based on deep belief nets (DBNs) network. Through this model, music features can be deeply trained, and features with higher intensity can be extracted and applied in audio classification. Dai et al. (2016) used long short-term memory (LSTM) neural network in audio classification, which provides a good solution for the processing of long-time series features, such as audio or speech signals. For many years, the research on LSTM neural networks has not stopped. In order to make the network to have a better processing ability for long sequence data, researchers have put forward many improved methods. Fulzele et al. (2018) fused LSTM and support vector machine (SVM) algorithms to classify music categories. The fusion model consists of two parts: firstly, correlation processing is carried out on the original audio and its features are extracted, and then these feature vectors are arranged and input into the two models, respectively. After training in the two models, the output data are fused. The experimental results show that the classification accuracy of the fusion of the two models is higher than that of the single model.

Huang et al. (2017) proposed a deep emotion representation model based on convolutional neural networks (CNNs) and LSTM networks. The model takes the processed data as the input, then uses CNN to obtain the feature vector of the data, LSTM receives the eigenvalues from CNN, and finally extracts the feature relationship between context and context through a cyclic neural network for emotion classification and prediction. In the same year, Badshah et al. (2017) proposed an audio emotion recognition model based on the spectrogram and deep CNNs (DCNNs). An important work of the model is to extract the spectrogram data of audio information first. The network structure is composed of three convolution functions and three fully connected functions, and audio recognition is carried out by obtaining spectrogram features. Due to the disappearance of the gradient when CNN deals with timing problems, the classification results do not meet the expectations.

Dong (2018) used a CNN for audio analysis. They preprocessed the initial audio data to obtain spectrogram information and directly asked CNN to learn this spectrogram information. This method still showed the ability to obtain timing information. In the same year, Lv et al. (2018) classified the audio based on the traditional SVM method, which verified that the traditional method still remained effective in audio processing. Hu et al. (2018) proposed a model method for the emotion classification of Chinese pop music. The author used the factor analysis (FA) algorithm to optimize radial basis function parameters and developed a modeling method of FA-SVM, so as to improve the performance of SVM.

In 2019, the attention mechanism model was popularized and applied to different depth learning methods. Yu et al. (2020) used the attention mechanism together with bidirectional recurrent neural network (BRNN) in the music category classification method. The model makes full use of the different characteristic information between the spectra. At the same time, the author conducted a comparative experiment between serial attention and parallel attention in the article. The experiment shows that the flexibility of parallel attention is better than that of serial. Xie et al. (2019a) proposed an attention-based LSTM classification model for audio emotion classification. Through the adjustment of weight, the model makes a good distinction between the information differences between each network layer, avoiding the information interference between each layer, especially the underlying network with more redundant information. In the same year, Xie et al. (2019b) extracted frame features from audio waveform information and used frame features to replace traditional speech features for audio recognition, because speech frames can well express the time-series relationship of speech, and more complex feature problems can be handled through these time-series relationships, they also put forward two improved methods: firstly, the forgetting gate in LSTM network is modified to improve the overall computing power of the model. The improved forgetting gate is only related to the previous information and has nothing to do with the information input at the current time point. Secondly, they added the attention mechanism to divide the weight of the local features of the model. Experiments proved that the model showed good performance in audio processing. Chen (2019) proposed an audio emotion classification method based on CNN-LSTM, which uses the fusion feature to achieve a better classification effect than a single feature. Lca et al. (2020) used the random forest method to classify speech emotion and proposed a classification algorithm based on two-layer fuzzy multiple random forest. This is a common clustering algorithm, which can divide high features and then become a subclass feature that is easier to calculate. This subclass feature uses the random forest for classification, showing a good classification effect. Bayu et al. (2019) proposed a speech emotion analysis model based on SVM + K-near neighbor (KNN), which is based on traditional machine learning. The KNN algorithm can detect linear or non-linear data and process different information. The author proved that this dimension method has certain advantages in speech classification. Orjesek et al. (2019) proposed a classification model based on the combination of CNN and recurrent neural network (RNN). The model directly takes the original audio signal as the input of the model. Experiments show that CNN combined with RNN can play a good performance in dealing with audio problems. Chen et al. (2019) proposed a multimodal emotion classification model. Experiments show that the recognition efficiency of this model is higher than that of a single model.

Liu and Tan (2020) used the multimodal classification method to classify audio, extracted different audio features in the experiment, and fused these features into the model for training. The experiment shows that the final classification accuracy of the model needs to be further improved. Zhang et al. (2020) proposed an audio emotion classification algorithm based on DCNN. The author first extracted the Mel feature map of speech, and then input it into the CNN network to extract and train the Mel spectrum. The model can accurately recognize most categories, but there is still the problem of inaccurate classification of some emotions. Cunningham et al. (2020) proposed a method of audio emotion recognition based on supervised learning, but the training ability of this method to time series samples remains to be verified. Wang G. et al. (2020) used an SVM multi-classification algorithm for emotion recognition of Chinese speech data, but only used Chinese speech data, did not make the experimental comparisons on more audio data, and did not verify the effectiveness of the algorithm in processing long-time series features.

To sum up, the audio emotion classification method based on deep learning has made a series of breakthroughs. CNN can accurately obtain the eigenvalues of audio information, and RNN can process the sequence information of audio. However, there are still some problems in the current methods, which are as follows: (1) for some complex linguistic spectrum, emotional features cannot be effectively recognized; (2) when processing audio sequence features, there will be problems, such as feature value loss. This paper will study the above problems that are existing in the current algorithm and propose a better emotion classification model for the automatic emotion recognition of Qin opera, so as to promote the artistic inheritance and cultural emotion color dissemination of Qin opera.

## Improved Convolutional Neural Network

With the continuous development of deep learning, a large number of studies have gradually used a neural network to replace the traditional acoustic model (Dahl, 2010). This paper will also use the latest research method to study the emotional classification of Qin opera. Among them, CNN is one of the earliest neural network architectures (Fukushima, 1980) and has been used to process audio information for a long time. Abdel Hamid proposed the application of a CNN in audio recognition 10 years ago (Abdel-Hamid et al., 2014), but there were still many problems in using the convolutional network to process audio information because CNN was not developed to a deep number of layers at that time. Later, the emergence of deep networks, such as visual geometry group net (VGGNET) and residual network (ResNet), greatly strengthened CNN’s ability to process audio information.

Convolutional neural network classification needs to input the original data and output the target (Lawrence et al., 1997). After inputting the audio data into the model, specify the sample value of the input song by calculating the characteristics of the original data, and finally, get the training data. Once the classification of the training data is determined, the test can be carried out to get the results.

In general, the spectrogram of audio is one of the key feature signals in audio processing. The spectrogram features contain important audio emotional information, but the audio signal has complex characteristics, such as feature diversity and environmental diversity. How to overcome these problems is the key to improve the accuracy of audio recognition.

Convolutional neural network has a convolution layer and pooling layer. This unique network structure makes it very easy to automatically extract local features. At the same time, it does not need a too complex operation process in training, which reduces the complexity of network training. The application of CNN in audio recognition uses the stability of the convolutional network model to overcome the diversity of audio features, so as to improve the efficiency of the whole training process. CNN has a very mature application in image processing, so it is very appropriate to use CNN to process the spectral features of audio, which has also been effectively verified in various experiments. However, there is still much room for improvement for the network itself and the characteristic information of data, which will also be an important work of speech analysis in the future.

In this paper, the CNN is deeply optimized, and the ResNet model is used to enhance the fitting ability and stability of the model. Combined with the attention mechanism, the expression of each weight information is strengthened, so that the network has a better ability to distinguish the characteristic information, so as to improve the overall performance of the network. Finally, through each group of experiments, the results of this model on the emotional classification of Qin opera are verified.

### Extraction and Processing of Language Spectrum Feature

In this paper, the audio signal is converted into map information in some way, and the map information is used as the input for network training. Because the spectrum contains rich emotional information in audio, this paper will extract the audio spectrum as the input data of the model.

The spectrogram shows the audio feature information in the form of a two-dimensional image (Mellinger and Clark, 2000). A spectrogram consists of two coordinates and is represented by the relationship between time and frequency. The frequency of audio reflects some speech features, such as pitch, spectral peak, and formant of audio information, and the energy value of the audio signal is represented by the depth of different colors. A spectrogram contains a large number of audio signal features.

In this way, the rules in the audio signal can be found according to the frequency distribution on the spectrogram and the change of energy value, and the emotional judgment of audio can be carried out according to these rules.

**FIGURE 1:**
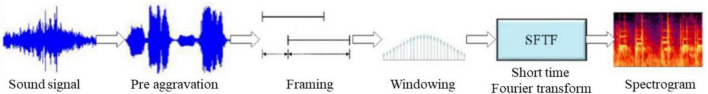
Spectrogram processing.

A complete spectrogram processing flow is shown in Figure 1. In the spectrogram, colors represent amplitudes, usually dark blue corresponding to low amplitudes, while brighter colors, such as red, represent gradually increasing amplitudes.

#### Preprocessing

The original signal characteristics of audio are very messy, which is not conducive to the effective implementation of classification experiments. Preprocessing is to better show the characteristics of audio signals. Common operations include denoising, aggravation, framing, and Fourier transform.

##### Denoising

Denoising is generally the first step of audio processing. There are many noises in the audio signal, such as environmental noise, high-frequency, and low-frequency noise, which often interfere with some subsequent processing, so denoising is an essential step in audio processing. Common denoising methods include filter denoising, wavelet transform, least mean square algorithm, and so on. This paper will introduce a method to reduce the noise of audio signals through threshold function. The core of this method is to operate the wavelet coefficients obtained from the original signal processing through a threshold function and then transform the results into the audio signals. In this way, other noise signals that affect the signal characteristics will be removed, and the signal characteristics will be further strengthened.

##### Preaggravation

When the high-frequency information is too weak, the whole feature is not easy to process. In order to obtain better feature information, preaggravation is needed to strengthen the high-frequency information, and a preaggravation filter is used for signal emphasis. Preaggravation is mainly used to balance the spectrum information, make the value more accurate, and improve the signal-to-noise ratio, which will make the Fourier transform more stable.

##### Farming

Framing is to divide a long-time frame signal into shorter frames. Because the audio characteristics are unstable, if the signal time is too long, it may cause a partial loss of information after the Fourier transform, which will lead to incomplete processing information. In order to strengthen the integrity of information, the audio signal is divided into short-time frames before Fourier transform, i.e., to reduce the length of each input signal and ensure that the frequency and amplitude of the speech signal are stable within this length, and then it is still stable information after Fourier transform.

In most cases, the length of each frame is set to 20–40 ms. In this paper, 25 ms are taken as a frame. However, in order to avoid the loss of the original signal after framing, a buffer part should be maintained between each signal frame. In this paper, the buffer size is set to 15 ms, which is the frame shift.

##### Windowing

After framing, the signal will still fluctuate, so the result generated by the Fourier transform is still unstable, which will eventually affect the correct expression of spectrogram. Windowing is to make both ends of the signal smoother. This paper will use Hamming window to add windows.

##### Fourier Transform

The above processing is to obtain a relatively stable result after Fourier transform and avoid errors, resulting in the loss of key information of the spectrogram. The time-domain information is transformed into frequency domain information through the Fourier transform, which is the core of the Fourier transform. In this paper, the short-time Fourier transform is used to process each frame signal. This transform is the result of a superposition of some short-time stable signals.

#### Generation of Spectrogram

After Fourier transform, the spectrogram of the corresponding audio signal can be generated. The audio signal matrix is expressed as:


(1) $$X=\left[X_{1}\,\ldots \,X_{M}\right]\in R^{N*M}$$

where N represents the length of the signal interval.

Finally, the generated spectrogram is as follows:


(2) $$L_{\log}\,\left(a,b\right)=20\,\mathrm{lg}\left(\left|\hat{X}\,\left(a,b\right)\right|\right)$$

where *L*_log_ is the spectrogram expression generated by signal X, *a* ∈ {1, 2, …, *A*} and *b* ∈ {1, 2, …, *B*} represents time and frequency information, respectively. Finally, the grayscale spectrogram is obtained by grayscale processing.

#### Optimizing Spectral Features

The generated spectrogram features are optimized to make the expressive ability of spectrogram features stronger. Firstly, the detailed features of the spectrogram are described by the filter; then the texture feature is extracted and fused with the improved histogram feature. Finally, the new feature is the optimized spectrogram feature. The filter function used in this paper is shown as:


(3) $$g\,\left(u,v\right)=\exp\left\{-\frac{\log{\left(u_{1}/u_{0}\right)}^{2}}{2\log{\left(k/u_{0}\right)}^{2}}\right\}\,\exp\left\{-\frac{v_{1}^{2}}{2\,\sigma_{v}^{2}}\right\}$$

where *u*_1_ = *u cos* θ + *v sin* θ, *v*_1_ = *u sin* θ + *v cos* θ, θ indicates direction, *u*_*0*_ is frequency, and *k* is bandwidth. The selected values of different *k* correspond to one *k* value, so that *k*/*u* will not change, so as to keep the function stable.

The expression of Log-Gabor spectrum of the spectrogram is as follows:


(4) $${\hat{G}}_{u,v}\,\left(a,b\right)=f\,\left(a,b\right)*g_{u,v}\,\left(a,b\right)$$

where *f*(*a*, *b*) is the spectrum image, *g*_*u*,*v*_(*a*, *b*) is the Log-Gabor function, and the final spectrum ${\hat{G}}_{u,v}\,\left(a,b\right)$ is obtained by convoluting the spectrum image with the Log-Gabor function. Here, *u* ∈ {0, 1, …, 4} and *v* ∈ {0, 1, …, 7}.

The optimized spectrogram reduces the dimension of features and increases the recognition ability of the model to the overall features.

### Optimization of Network Structure

In order to optimize the training ability of the network structure, this section will optimize the neural network. Mainly considering the model, the learning rate of the network, the number of network layers, and the network itself, the network model suitable for this paper will be found through different experiments.

#### From the Perspective of the Model

There are four improved methods, weight attenuation, dropout, batch regularization, and local response normalization (LRN), from the perspective of the model.

##### Weight Attenuation

This method can effectively deal with the over fitting problem by adding regularization to the original data. The regularization term is as follows:


(5) $$C=C_{0}+\frac{\gamma }{2\,n}\,\sum w^{2}$$

where *C*_*0*_ is the original data, n is the sample size, and coefficient λ is to balance the weight. The complete L2 regularization process is to square and sum all the parameters and divide them by n.

##### Dropout

The process of network training will consume a lot of time, and dropout is used to avoid fitting phenomenon in training. The training process of the standard model is as follows: first input the data for propagation, get an error, back-propagation, and then update the parameters. After referring to dropout, ensure that the input and output remain unchanged, delete some local hidden information, update the value after two-way propagation, and then cycle the following process: restore the deleted neurons, return to the previous steps, and repeat them in turn until the whole training is completed.

##### Batch Regularization

With the increasing depth of the network, its convergence speed will be slower and slower. In order to improve the training speed of the whole network, the data are processed in batches to keep the data evenly distributed. Batch regularization can avoid the problem that no data activate neurons or all data are involved in activation. The steps of batch regularization are as follows:

Calculate mean value:


(6) $$\mu \,\beta \leftarrow \frac{1}{m}\,\sum_{i=1}^{m}x_{i}$$

Calculate data variance:


(7) $$\sigma_{\beta }^{2}\leftarrow \frac{1}{m}\,\sum_{i=1}^{m}{\left(x_{i}-\mu \,\beta \right)}^{2}$$

Normalization:


(8) $${\hat{x}}_{i}\leftarrow \frac{x_{i}-\mu \,\beta }{\sqrt{\sigma_{\beta }^{2}+\varepsilon }}$$

Scale shift:


(9) $$y_{i}\leftarrow \gamma \,{\hat{x}}_{i}+\beta =B\,N_{\gamma \,\beta }$$

##### Local Response Normalization

This processing can change the value of neurons in the network and improve the training speed of the whole model. The LRN expression is shown as:


(10) $$b_{x,y}^{i}=\frac{a_{x,y}^{i}}{{\left(k+\alpha \,\sum_{j=\max\left(0,i-\frac{n}{2}\right)}^{\min\left(N-1,i+\frac{n}{2}\right)}{\left(\alpha_{x,y}^{j}\right)}^{2}\right)}^{\beta }}$$

where α indicates output, $a_{x,y}^{i}$ represents location information, which has four parameters [a, b, c, and d] where a is the picture sequence, b is the height, c is the width, and d is the channel ordinal number. N is the number of channels and n is the nearest nucleus in the same position. k, n, α, and β are generally set to fixed values, such as k = 2, n = 5, α = 1 × e − 4 and β = 0.75.

#### From the Perspective of the Learning Rate of the Network

Learning rate, also known as step size, determines the convergence speed of the model. Too large or too small learning rate will affect the performance of the model. This paper attempts to adjust the learning rate to an optimal value in the experiment. The specific steps are as follows: set a large initial value for the learning rate, observe the experimental results, especially the accuracy and the value of the objective function, and get the best result through continuous adjustment. Among them, the learning rate can be expressed in two forms, as shown in equation (11) in exponential form and equation (12) in fractional form.


(11) $$\alpha =0.95^{e\,p\,o\,c\,h\,\_\,n\,u\,m}\,\alpha_{0}$$

(12) $$\alpha =\frac{\alpha }{1+d\,e\,c\,a\,y\,\_\,r\,a\,t\,e\cdot e\,p\,o\,c\,h\,\_\,n\,u\,m}$$

The change range of learning rate needs many experiments and finally gets the best learning rate suitable for this model.

#### From the Perspective of the Number of Network Layers

For neural networks, different depths will obtain different eigenvalues. Generally, the underlying network extracts the underlying features. With the increase of the number of network layers, the performance of the features becomes stronger. Therefore, deepening the number of network layers is also an effective method to improve the performance of the model. However, the relationship between model performance and network depth does not increase exponentially. When the network deepens to a certain depth, the model performance will reach an optimal value, and then the performance will decline. Therefore, it is very important to select an appropriate network depth.

#### From the Perspective of Network

**FIGURE 2:**
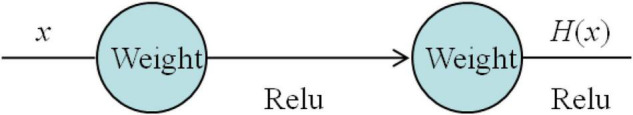
General neural network.

As mentioned above, increasing the number of network layers may cause the problem of network degradation. If you want to continue to optimize the model, you need other ways. The introduction of the ResNet solves the problem of network depth. Figure 2 shows the form of a general neural network. Each layer has a weight and activation function. The network uses the rectified linear activation function (Relu) activation function.

**FIGURE 3:**
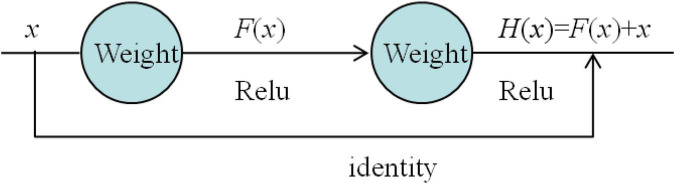
Residual network model.

The residual model is shown in Figure 3.

As can be seen from Figure 3, the network model has one more identity connection than the general neural network, which is directly connected from the input to the front of the output. Except for the identity of this connection, it is no different from the form of the conventional neural network module. The input *x* is superimposed by two weights and activated by the Relu function, but it is such a “shortcut” that makes the “general” network no longer general.

Assuming that x is the fitting function and (*x*) is the value of a certain data, the so-called residual is a difference between (*x*) and *x*, i.e., (*x*). The representation of the residual element is as follows.


(13) $$y_{l}=h\,\left(x_{l}\right)+F\,\left(x_{l},W_{l}\right)$$

(14) $$x_{l+1}=f\,\left(y_{l}\right)$$

where *x*_*l*_ represents input and *x*_*l+1*_ represents output. They all represent values in the same unit. The residual unit is composed of multiple layers, and *f* is the Relu function. A shallow to deep eigenvalue *x*_*L*_ is obtained through calculation, as shown below.


(15) $$x_{L}=x_{l}+\sum_{i=1}^{L-1}F\,\left(x_{i},W_{i}\right)$$

Different from the traditional CNN network, the calculation method of the ResNet has changed, and the depth of the network is not deepened by increasing the number of layers. According to the principle of backpropagation, the cost function is ε. If the partial derivative of *x*_*l*_ is obtained, there is equation (16).


(16) $$\frac{\partial \varepsilon }{\partial x_{l}}=\frac{\partial \varepsilon }{\partial x_{L}}\,\frac{\partial x_{L}}{\partial x_{l}}=\frac{\partial \varepsilon }{\partial x_{L}}\,\left(p+\frac{\partial }{\partial x_{l}}\,\sum_{i=1}^{L-1}F\,\left(x_{i},W_{i}\right)\right)$$

where coefficient *p* takes 1. If it is greater than 1 or less than 1, it will cause common gradient problems. Therefore, when the value is 1, the gradient can be propagated correctly.

To sum up, in the ordinary CNN, when the number of layers becomes more, the forward propagation information will disappear from the bottom to the top, and the error from the top to the bottom is also difficult to propagate in the backpropagation. In this way, some problems will appear, making the accuracy after training inaccurate. The ResNet adds a connection path on the basis of the ordinary neural network, which effectively transmits the data to the next layer without causing problems, such as disappearance, and then transmits it to the deeper layer by layer, which is the characteristic of the ResNet. Residuals are the reinforcement of subtle changes.

**FIGURE 4:**
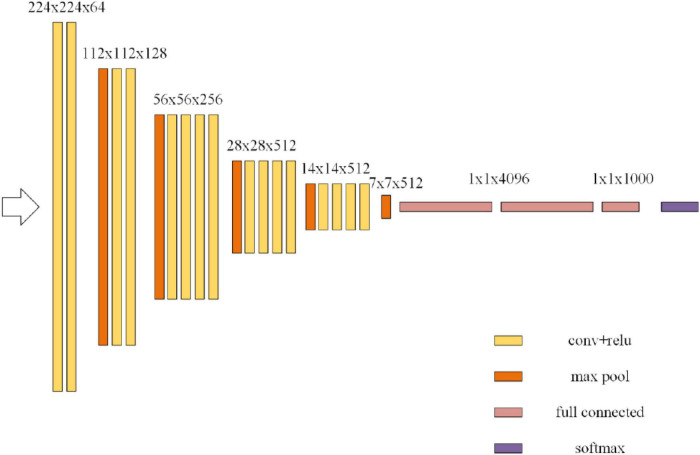
Visual Geometry Group (VGG)-19 network.

The ResNet is improved on the basis of Visual Geometry Group (VGG)-19 network. The ResNet first extracts the bottom features and then extracts the deepest features. Each layer combines these features for judgment and output. This method makes full use of each layer of information to avoid the loss of features in the training process. Figure 4 shows the VGG-19 network structure.

**FIGURE 5:**
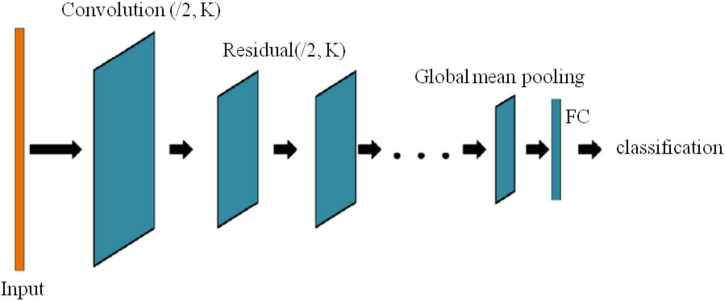
Multi-head attention residual network model.

Figure 5 shows a depth residual shrinkage network, where K is the number of convolution kernels and the step size is 2.

## Multi-Head Attention Residual Network Model

### Attention Model

The attention model of the machine is similar to a way of screening information. When we see a thing, the brain will focus on specific information about the thing, and some information that the brain thinks is not important will be selectively discarded. In fact, the brain is to analyze the observed things, sort the importance of information accordingly, focus on some things by controlling the brain’s thinking, and then ignore others (He et al., 2016). This process is actually a set of attention mechanisms of the human brain.

Similarly, we can apply this mechanism to the machine and add a certain degree of weight to the information. The important information machine will assign more weight and the less important information will assign less weight. In this way, the machine assigns different weights to different output information, so as to achieve focused selection, so as to speed up the operation speed of the model and increase the recognition rate of the model.

The essence of the attention mechanism is to imitate the attention function of the human brain, filter useful information, and filter useless information. It is an effective information distribution method to allocate important tasks to needed resources. This method was first applied in machine translation and has developed rapidly, especially widely used in solving related problems, such as machine translation, audio recognition, emotion analysis, text tagging, and so on. This is because it is proved to be very effective to solve these problems through the attention model.

In the computer, attention is used to calculate the weight of the current output value and divide the weight. The attention algorithm process is described below.

It is assumed that an input sequence is as (*h*_1_,*h*_2_,…,*h*_*n*_). The output and state of each sequence are matched to obtain the matching vector of each sequence as follows.


(17) $$e_{i\,j}=a\,\left(S_{i-1},h_{j}\right)$$

The corresponding weight distribution of each sequence is obtained.


(18) $$\alpha_{i\,j}=\frac{\exp\left(e_{i\,j}\right)}{\sum_{k=1}^{T_{X}}\exp\left(e_{i\,k}\right)}$$

Thenα_*ij*_ is weighted and summed to obtain the input *c*_*i*_of the next sequence:


(19) $$c_{i}=\sum_{j=1}^{T_{X}}\alpha_{i\,j}\,h_{j}$$

Finally, the output result is calculated:


(20) $$S_{i}=f\,\left(S_{i-1},y_{i-1},c_{i}\right)$$

The above is the complete processing steps of the attention mechanism. After such processing, the important information in each sequence can be screened out and then transmitted to the next sequence. The final result will also retain the key information of each sequence, so as to prevent the loss of information and improve the overall performance of the model.

**FIGURE 6:**
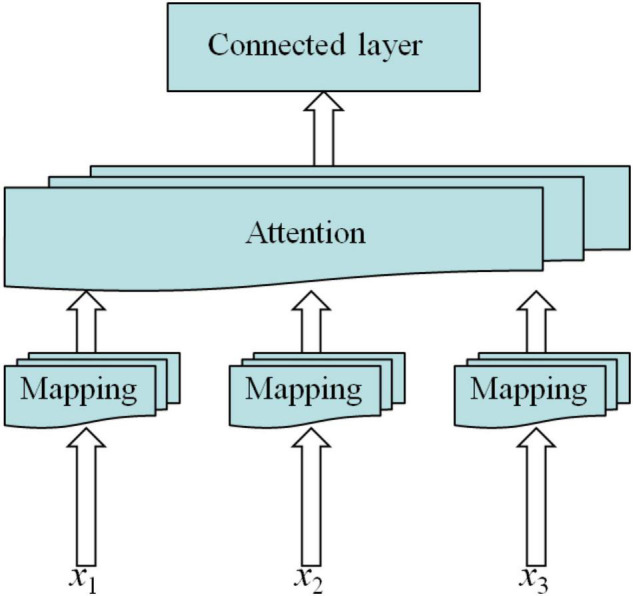
Multi-head attention mechanism.

The multi-head attention model was first proposed by the Google team in 2017. Multi-head attention is improved on the basis of the attention model (Jian et al., 2021; Yun et al., 2021). For the input of different parts of information, multiple queries of multi-head attention mechanism are used to transfer this information to different internal spaces of the model. The values of each space are calculated and spliced together. Compared with the single attention mechanism, multi-head attention can learn useful information in neurons from a variety of angles and has better interpretability for long-time series of information (Lin et al., 2020; Wang J. et al., 2020; Chen et al., 2021). The structure of multi-head attention model is shown in Figure 6.

### ResNet With Multi-Head Attention Mechanism

The multi-head attention mechanism is introduced in the model and a multi-head attention ResNet, namely, MHAtt_ResNet, is proposed. The model first inputs the extracted spectrogram into the ResNet for training, then adds multi-head attention to the network, calculates the correlation between each query and each key value through attention and normalizes it to obtain the weight distribution, and adds these weights to obtain a set of vectors. The specific calculation process is as follows.


(21) $$a_{n}=s\,o\,f\,t\,m\,a\,x\,\left(\frac{Q\,K^{T}}{\sqrt{d_{k}}}\right)\,V$$

(22) $$a\,t\,t\,\left(\left(K,V\right),Q\right)=a\,t\,t\,\left(\left(K,V\right),q_{1}\right)⊕\ldots ⊕a\,t\,t\,\left(\left(K,V\right),q_{n}\right)$$

The vector output from the attention layer is calculated by *softmax* to obtain the final result.

**FIGURE 7:**
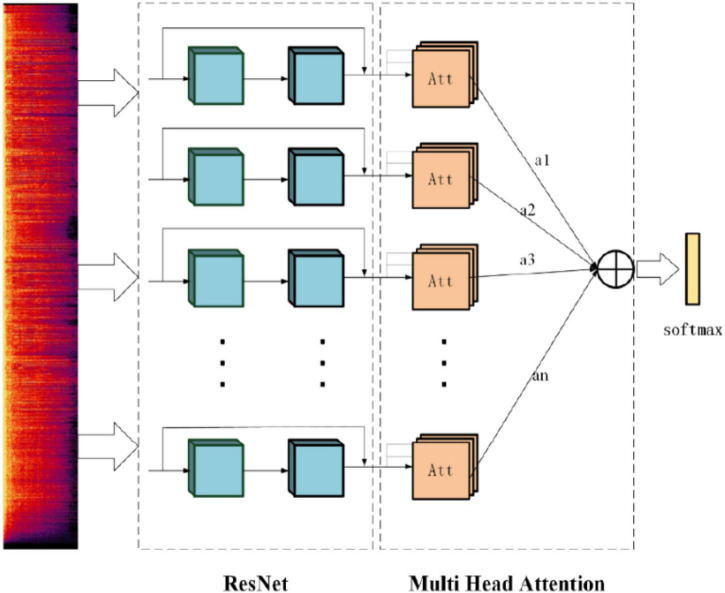
Multi-head attention residual network model.

The model structure is shown in Figure 7, which is divided into the input layer, ResNet layer, and attention layer. Among them, the attention layer scales the weight, which not only prevents the redundancy of calculation results caused by too high dimension but also deepens the relationship between distant information in a long-time series.

This model (Cazenave, 2018; Zhang K. et al., 2018) has the following advantages:

(1) It can reduce the loss of important information;
(2) Compared with the general CNN network, it is more suitable to deal with long-time series problems; and
(3) Multiple independent attention operations effectively prevent over fitting.

## Experiment and Analysis

### Data Set

This paper will use WebFace dataset (CASIA) data and Qin opera data for experimental comparison. CASIA data are Chinese emotion data sets recorded by professional researchers in a research institute in China that includes 9,600 different pronunciations, i.e., six emotions: neutral, happy, angry, sad, afraid, and surprised. The data set shows the sound characteristics of various emotions and has a high experimental value for speech emotion analysis. Part of the data set is the same content, but it is endowed with different emotional colors by different recorders, which allows the machine to learn the ability to process different emotional speech information and sound features under the same text. In this paper, 300 different emotional data with the same content are selected for slicing and other preprocessing. The audio sampling frequency is set to 16 KHz, one segment is segmented after every 30 s, there are a total of 1,200 voice segments, and the number of frames of each segment is 1,620. The slicing data are divided into training set and test set according to the ratio of 8:2. Qin opera data contain a variety of materials, such as myths and folk stories, which have rich emotional colors. All Qin opera data used in this experiment are the original audio collected on the network, a total of 800 tracks are collected, and they are preprocessed, such as segmentation. It is also a segment of every 30 s, with a total of 5,000 segments, which are divided into training set and test set according to the ratio of 8:2. In order to make an effective experimental comparison with the emotions of CASIA data, Qin opera is still divided into six emotions: happiness, neutrality, anger, sadness, fear, and surprise.

### Evaluation Criterion

For the classification problem, we need to find an index to measure the final classification result, in which the precision (P), recall (R), and F1 are a set of appropriate evaluation indexes. These three indexes will also be used to evaluate the experimental results of the article.

The precision rate refers to how much of the predicted correct data is indeed correct, so it is also called the accuracy rate, and the recall rate is also called the recall rate, indicating the proportion of the predicted correct data in all the correct data; F1 value is harmonic averaged based on the first two values, and it pays more attention to the one with small value. Among them, P1, P2, N1, and N2 represent real cases, false-positive cases, true counterexamples, and false counterexamples, respectively.

The calculation of the three indicators is as follows.


(23) $$p\,r\,e\,c\,i\,s\,i\,o\,n=\frac{P\,1}{P\,1+P\,2}$$

(24) $$r\,e\,c\,a\,l\,l=\frac{P\,1}{P\,1+N\,2}$$

(25) $$F\,1=\frac{2\cdot p\,r\,e\,c\,i\,s\,i\,o\,n\cdot r\,e\,c\,a\,l\,l}{p\,r\,e\,c\,i\,s\,i\,o\,n+r\,e\,c\,a\,l\,l}$$

### Result Analysis

#### Analysis of Experimental Results of CASIA Data Set

The algorithm in this paper (MHatt_ResNet) is compared with the multi-level residual CNN model and CNN network (Zeng and Zhang, 2018; Wang et al., 2021) and evaluated with different indicators. The comparison results are shown in Tables 1, 2. Table 1 lists the precision (P), recall (R), and F1 values of different algorithms, and Table 2 shows the classification accuracy of each emotion by three methods.

**TABLE 1:** The recognition accuracy, recall, and F1 value of CASIA data set by different methods (%).

	*P*	*R*	F1
CNN	55.48	56.88	56.64
Multi-level residual CNN	65.22	64.02	64.28
MHatt_ResNet	71.22	72.29	72.35

**TABLE 2:** The accuracy of emotion classification by different methods (%).

	Happiness	Neutrality	Anger	Sadness	Fear	Surprise	Accuracy
CNN	54.52	53.20	64.27	54.84	52.03	61.83	55.19
Multi-level residual CNN	66.21	59.86	66.03	65.24	64.77	62.57	64.03
MHatt_ResNet	80.25	65.22	69.71	72.08	65.87	77.05	72.39

It can be seen from the data in Tables 1, 2 that when the same data set is used, the performance of the CNN model is the worst and its classification accuracy is the lowest. This is because a single network has poor processing ability of spectral information and cannot read key emotional information. The algorithm (MHatt_ResNet) proposed in this paper is obviously better than the other two algorithms. Experiments show that the CNN model with multi-head attention mechanism can better identify emotional information, and the classification accuracy is significantly improved.

The spectrogram as the input feature of the model is extracted. The spectrogram contains a large number of audio emotional signal features. Its frequency distribution and energy value change law can well express the different emotional characteristics of different audio. Compared with other features, the spectrogram is an audio feature more suitable for emotion classification. This paper will compare the effects of different features on audio emotion classification results through experiments to verify the advantages of spectral features as model input in emotion classification. Table 3 shows the comparison of the accuracy of emotion classification by different features.

**TABLE 3:** Comparison of classification accuracy of different audio features.

	Happiness	Neutrality	Anger	Sadness	Fear	Surprise	Accuracy
MFCC	59.66	47.52	61.03	63.23	55.86	62.02	58.55
Spectral Centroid	64.22	55.88	63.52	64.58	60.21	61.55	60.53
Spectrogram	79.22	64.02	65.25	72.58	66.54	71.01	70.29

The experimental results show that different features as model input will produce different classification results. Among them, the mel frequency cepstral coefficients (MFCC) feature has the worst effect when compared with the other two features in emotion classification. Spectrogram can accurately represent emotional information. Taking spectrogram as feature input makes the model to have the highest accuracy for all kinds of emotion recognition. Through the comparison of the recognition accuracy of various emotions, it can be seen that no matter which feature is used, the recognition degree of happiness and sadness is very high. This is because the expression of these emotions is more prominent, and it is easier for computers to recognize them. Experiments show that this paper takes spectrogram as feature input and has better recognition accuracy than other features.

#### Experimental Result Analysis of Qin Opera Data

In this group of experiments, Qin opera data and CASIA data are used to compare the classification accuracy. The two data are trained with the proposed model, and the classification accuracy of different emotions is shown in Table 4.

**TABLE 4:** Comparison of classification accuracy of two kinds of data.

	Happiness	Neutrality	Anger	Sadness	Fear	Surprise	Accuracy
CASIA	79.33	63.52	68.97	73.22	65.94	71.21	70.65
Qin opera	83.02	62.51	72.23	72.89	70.23	71.02	71.82

Experiments show that the training results of the model using two kinds of data are very close, which shows that the model has good stability when dealing with different data. Among them, the model has the highest recognition rate of “happy” emotion in Qin opera data, while “neutrality” emotion is difficult to be recognized by computer because its definition is very vague.

**FIGURE 8:**
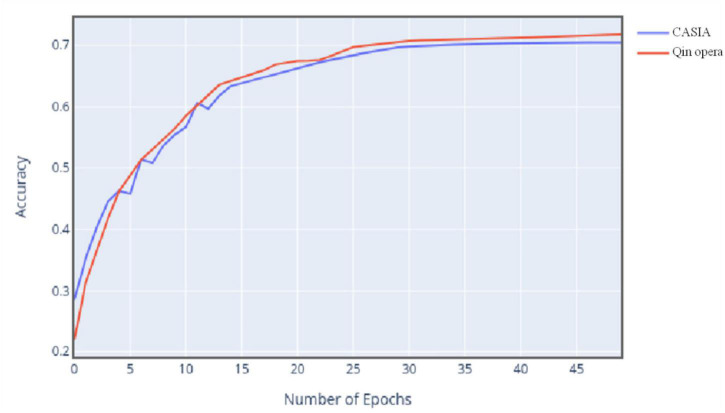
Change curve of classification accuracy of two data sets.

Figure 8 shows the change curve of classification accuracy of two data sets with the increase of iteration times.

The experimental results show that with the gradual increase of the number of iterations, the accuracy trend of the model for the two data is almost the same, but the accuracy of the final Qin opera data is slightly higher than that of CASIA data, because different sample numbers also have a certain impact on the accuracy. In this experiment, Qin opera data are larger than CASIA data, so the effect of model training is better. It can be seen that the size of data will also affect the model training process.

Through experiments, the multi-head attention ResNet model has high emotional classification accuracy for Qin opera, and with the increase of the number of data sets, the model will train a better classification effect. However, from the results, the accuracy of the overall classification still has a lot of room to rise. How to further improve the accuracy of Qin opera emotion classification is the main direction of future research.

## Conclusion

Qin opera is a traditional folk opera in China, which has been passed down for thousands of years. At present, due to the single expression of folk art, the younger generation is no longer enthusiastic about traditional culture, thus Qin opera is now facing the danger of no one can sing or even disappearing. Therefore, to spread the Qin opera, modern technology elements can be incorporated into Qin opera performances. For example, combining virtual reality technologies, such as VR display or immersive experience, with traditional Qin opera performances. The purpose is to realize diversified performance forms, which will give Qin opera a new life. In this way, traditional culture can be protected and inherited.

The emotions of Qin opera are complex and diverse, which make it is very difficult for a computer to automatically understand. Therefore, the problem of how to make the computer automatically recognize the emotions expressed in Qin opera is quite urgent to be solved. Currently, deep learning technologies are widely used in audio processing-related fields. But the existing models are not accurate enough in classifying long-time series data. Therefore, how to improve the model’s accuracy in Qin opera emotion recognition is the main research content of this article. A MHAtt_ResNet is proposed. The spectrogram features contain important emotional information, so this article uses spectrogram as the model input. Combines the ResNet and multi-head attention mechanism to make the model have stronger information recognition capabilities. The model proposed in this article can effectively avoid the loss of key emotional characteristics. The results show that the MHAtt_ResNet can effectively improve the accuracy of audio emotion classification.

Through the model training with CASIA data and the experimental comparison with the other two network methods, it is verified that the model in this paper is feasible in dealing with the problem of audio emotion classification, and the results are better than the other two models. Then the Qin opera data are tested and compared with the results of CASIA data. The experimental results show that the model has good stability. However, there are still some problems in the model. Due to some limitations of the convolution network in processing audio information, the extraction of timing information by the network is not very good, which will lead to the loss of features in the training process, resulting in a great improvement in the overall accuracy of the model. Therefore, the model can be further studied and improved, and it is necessary to get a better model to deal with audio timing characteristics.

## Data Availability Statement

Publicly available datasets were analyzed in this study. This data can be found here: https://download.csdn.net/download/weixin_44263873/12303282. Name: CASIA.

## Ethics Statement

The studies involving human participants were reviewed and approved by ethics committees of Northwest University and Xi’an Polytechnic University. Written informed consent for participation was not required for this study in accordance with the national legislation and the institutional requirements.

## Author Contributions

The author confirms being the sole contributor of this work and has approved it for publication.

## Conflict of Interest

The author declares that the research was conducted in the absence of any commercial or financial relationships that could be construed as a potential conflict of interest.

## Publisher’s Note

All claims expressed in this article are solely those of the authors and do not necessarily represent those of their affiliated organizations, or those of the publisher, the editors and the reviewers. Any product that may be evaluated in this article, or claim that may be made by its manufacturer, is not guaranteed or endorsed by the publisher.
